# Alginate-Based Biomaterials: From Fundamental “Egg-Box” Chemistry to Diverse Biomedical and Metabolic Management of Obesity and Diabetes

**DOI:** 10.3390/gels12030250

**Published:** 2026-03-17

**Authors:** Adnan Alsaei, Ahmad Zarwi, Ayah Binrajab, Fatema Rahimi, Renad AlAnsari, Manyam Praveen Kumar, Alexandra E. Butler, Stephen L. Atkin, G. Roshan Deen

**Affiliations:** 1 School of Medicine, Royal College of Surgeons in Ireland (RCSI), Medical University of Bahrain, Busaiteen 228, Bahrain; 23200222@rcsi.com (A.A.); 24204621@rcsi.com (A.Z.); 24200305@rcsi.com (A.B.); 23200122@rcsi.com (F.R.); 21200799@rcsi.com (R.A.); abutler@rcsi-mub.com (A.E.B.); 2 School of Postgraduate Studies & Research, Royal College of Surgeons in Ireland (RCSI), Medical University of Bahrain, Busaiteen 228, Bahrain; pmanyam@rcsi-mub.com (M.P.K.);

**Keywords:** alginate, hydrogels, biomaterials, egg-box model, medical applications, metabolic health

## Abstract

Alginate, a naturally occurring polysaccharide derived from brown algae, has emerged as a versatile cornerstone in the field of biomedical materials. Its widespread adoption is driven by its exceptional biocompatibility and the unique cation-dependent gelation defined by the “egg-box” model. This review examines the fundamental chemistry of alginate, detailing how its crosslinking mechanisms dictate the physicochemical properties essential for clinical performance. The discussion bridges the gap between polymer structure and diverse biomedical applications, including drug delivery, tissue engineering, and the clinical management of gastrointestinal reflux and wound care. Furthermore, the article evaluates the role of alginate-based systems in the biomedical and metabolic management of obesity and diabetes. By analyzing how alginate influences satiety, glycemic index modulation, and lipid absorption through biophysical mechanisms, this review highlights the transition from fundamental chemical architecture to practical clinical utility. By integrating structural chemistry with physiological impact, this work underscores the evolving potential of alginate-based materials as supportive and functional strategies in modern clinical care.

## 1. Introduction

Alginate is a natural polysaccharide obtained primarily from marine brown algae (Phaeophyceae class). This material has gained much popularity in biomedical research, such as targeted drug delivery, tissue engineering, 3D bioprinting, and heavy metal chelation, owing to its properties (natural abundance, minimal toxicity, biocompatibility, and film-forming attributes) and therapeutic potential (immunomodulatory, antioxidant, and antitumor effects) [1,2,3,4,5].

One of the main properties is its ability to form gels in the presence of divalent cations such as calcium or barium ions. This unique feature allows the formation of hydrogels at physiological conditions, which is vital for a variety of therapeutic biomedical applications [6]. In nanomedicine, alginates are applied in the form of emulsions, liposomes, solid-lipid nanoparticles, polymeric micelles, dendrimers, and nanocrystals [7]. Wound dressing based on alginate maintains a physiologically moist microenvironment, minimizes bacterial infection and promotes wound healing. Controlled and targeted release of drugs, proteins, and small molecules from alginate gels can be achieved through the interplay of alginate-based hydrogel chemistry. In addition, alginates act as a protective carrier for proteins, thereby minimizing the risk of denaturation [8]. In tissue engineering, alginate gels offer promise in cell transplantation and the development of implants.

Despite the numerous advantages of alginate gels in biomedical applications, they suffer from a few limitations in terms of permeability, degradability and mechanical properties [9,10]. However, these limitations have been addressed through optimization of extrinsic factors such as pH, temperature, and co-polymerization with other biopolymers [11]. This review aims to bring together the chemistry of alginates and their recent advances and future perspectives in therapeutic biomedical applications and the management of metabolic disorders.

## 2. Source and Chemistry of Alginate

Sodium alginate was first discovered by Kelp in 1883; it is an anionic hydrophilic heteropolysaccharide that is present in brown seaweed and as capsular polysaccharides of some soil bacteria [12]. The brown algae contain pigments such as chlorophyl a and c, carotene, and xanthophyl (pyroxanthin), and it is the xanthophyl that gives brown algae its distinct colour. The polysaccharides that are extracted from brown algae are alginates, fucoidans, and laminarans and are widely used in the biomedical and food industries [13,14,15].

**Figure 1:**
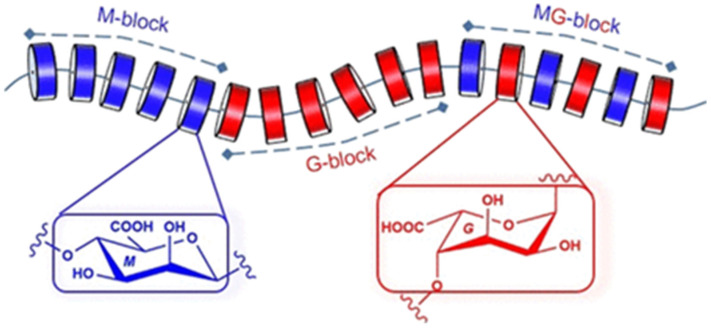
Schematic representation of the chemical structure of alginate highlighting the various blocks. Reprinted from ref. [16].

Alginate is a linear polysaccharide composed of two uronic acid monomers, viz., β-D-mannuronic acid (M) and α-L-guluronic acid (G). The ratio of M to G can vary depending on the source of alginate. The monomers are arranged in distinct blocks such as G-blocks, M-blocks and MG-blocks, as shown in Figure 1.

The distribution and the proportion of these blocks greatly influence the physical and chemical properties of alginates. M-blocks enhance the solubility but reduce the gel-forming capability, while the G-blocks provide rigidity and robust gel-forming capability. The MG-block with alternating M and G units contributes to elasticity and texture by providing a balance of rigidity and flexibility.

**Figure 2:**
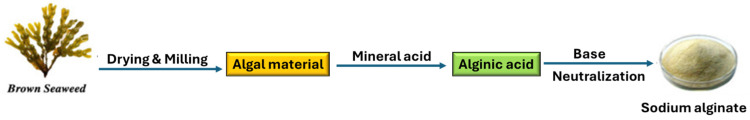
Illustration of purification of alginate from brown seaweed. Modified from ref. [17].

Commercial alginates are primarily sourced from various species of brown algae such as *Laminaria hyperborea*, *Laminaria digitata*, *Macrocystis pyrifera*, *Ascophyllum nodosum*, *Ecklonia maxima*, *Saccharina japonica* (formerly *Laminaria japonica*), *Lessonia nigrescens*, *Durvillea antarctica* and *Sargassum* spp. [17]. These algae thrive well in the coastal areas of Canada, Norway, Ireland and China. The extraction process involves acid treatment of brown algae with a mineral acid (hydrochloric acid) followed by alkaline treatment (sodium or potassium hydroxide). The alginate obtained after the chemical treatment is purified by precipitation using calcium chloride. The isolation process is illustrated in Figure 2.

The purity of alginates is very critical for biomedical applications as the impurities associated with raw alginates can provoke immune responses such as pathogen-associated molecular patterns (PAMP) and damage-associated molecular patterns (DAMP). Based on the source of brown algae and the extraction process, the molecular weight of alginate varies considerably. Commercially available alginates exhibit molecular weights in the range 3.2 × 10^4^ to 4 × 10^5^ g mol^−1^. An increase in molecular weight is associated with an improved gelation rate, viscosity, elasticity and tensile strength [18]. However, very high molecular weight is associated with extremely high viscosity, and this is not favourable for certain biomedical applications that involve immobilization of cells and drugs [19].

The solubility and viscosity of alginates are affected by various factors such as distribution of M- and G-blocks, degree of polymerization, temperature, ionic strength, pH and concentration. Alginate with a high content of G-blocks is more soluble in water, and the solubility increases with an increase in the pH of the solution. Under these conditions, the carboxylic acid groups (-COOH) are deprotonated (COO^−^) and repel each other due to coulombic interactions. This allows the blocks to remain well dispersed, thereby improving the solubility [20].

Comprehensive physicochemical characterization is essential to ensure reproducibility, safety, and performance of alginate across different natural and commercial sources. Because alginate is primarily extracted from brown seaweeds, variability in species, harvest conditions, and extraction protocols can significantly affect purity, molecular weight distribution, and monomer composition, all of which influence gelation behaviour and mechanical properties.

Purity assessment typically involves evaluation of residual proteins, polyphenols, endotoxins, and inorganic contaminants originating from the raw biomass and extraction process. Ash and moisture content analyses are routinely performed to determine inorganic residue and storage stability. Trace metal quantification, particularly of calcium, magnesium, and heavy metals, is commonly conducted using inductively coupled plasma (ICP) techniques, as these ions may influence crosslinking behaviour or raise safety concerns in biomedical applications. Structural verification of the polysaccharide backbone is generally performed using Fourier-transform infrared spectroscopy (FTIR), while solution-state nuclear magnetic resonance (NMR) spectroscopy remains the standard method for determining the mannuronic acid (M) to guluronic acid (G) ratio and sequence distribution [21,22].

However, solution-state NMR presents limitations for highly viscous systems or insoluble crosslinked materials. In such cases, solid-state NMR techniques, particularly ^13^C cross-polarization/magic angle spinning (CP/MAS), provide valuable atomic-level information on monomer composition, block distribution, and structural modifications induced by ionic crosslinking. Gel-state NMR approaches further enable investigation of polymer chain mobility, water–polymer interactions, and ion coordination dynamics within hydrated networks. These techniques are particularly informative for elucidating structural rearrangements associated with the “egg-box” model of calcium-mediated gelation [23].

Accurate determination of molecular weight and molecular weight distribution is equally critical, as these parameters strongly affect viscosity, gelation kinetics, and mechanical strength. Size-exclusion chromatography (SEC), often coupled with multi-angle light scattering (MALS) or refractive index detection, is the most widely reported method for determining number-average (Mn) and weight-average (Mw) molecular weights, as well as dispersity (Đ). Given the polyelectrolyte nature of alginate, careful control of ionic strength and solvent conditions is necessary to minimize aggregation during analysis. Dynamic light scattering (DLS) may be used to estimate hydrodynamic size in dilute solution, although it provides indirect molecular weight information and is sensitive to aggregation phenomena. Matrix-assisted laser desorption/ionization time-of-flight mass spectrometry (MALDI-TOF) can offer insight into oligomer distributions and lower molecular weight fractions, though its applicability to high-molecular-weight, highly polydisperse samples may be limited [19,20].

To enhance comparability among commercial alginates derived from different algal species or processing methods, it is recommended that studies clearly report extraction conditions, purification steps, analytical methodologies, calibration standards, and whether M_n_, M_w_, and dispersity values are provided. Routine batch-to-batch characterization of molecular weight distribution, M/G ratio, viscosity, and contaminant levels is particularly important for biomedical and pharmaceutical applications, where regulatory compliance and reproducibility are critical.

## 3. Mechanism of Gel Formation

### 3.1. Ionic Crosslinking

**Figure 3:**
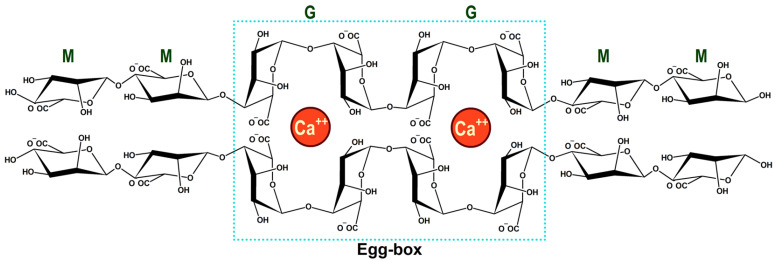
Illustration of ionotropic crosslinking of alginate and formation of the egg-box structure.

Alginates form gels through ionotropic crosslinking with various metal cations. The process of ionic crosslinking involves the formation of ‘egg-box’ structures in which the metal cations are coordinated between adjacent alginate chains. The process of crosslinking with a divalent metal ion is shown in Figure 3 [24].

The type and valency of metal ions affect the properties of alginate gels. Divalent cations (Ca^2+^, Cu^2+^, Ba^2+^, Sr^2+^) of calcium, copper, barium and strontium, and trivalent cations (Fe^3+^, Al^3+^) of iron and aluminum, can effectively crosslink with alginate G-blocks through coordination with carboxylate and hydroxyl groups [25]. The binding strength and crosslinking density depend on the charge and ionic radius of the metal ions, which greatly influence the mechanical and swelling properties of the gel.

Ionic crosslinking of metal ions with different blocks on alginate chains has been explored since the 1970s [26]. The following affinity series have been recognized [27,28]: (i) G-blocks: Pb^2+^ > Cu^2+^ > Cd^2+^ > Ba^2+^ > Sr^2+^ > Ca^2+^ > Co^2+^, Ni^2+^, Zn^2+^ > Mn^2+^; (ii) M-blocks: Ba^2+^ > Sr^2+^~Ca^2+^; (iii) MG-blocks: Ca^2+^ > Sr^2+^~Ba^2+^.

### 3.2. Covalent Crosslinking

**Figure 4:**
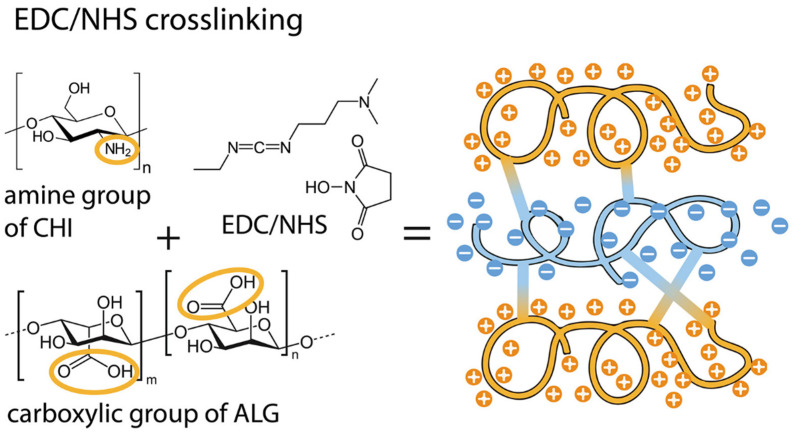
Illustration of carbodiimide chemistry and formation of stable amide bonds in alginate–chitosan gels. Image reprinted from ref. [29].

Covalent crosslinking involves the formation of permanent chemical bonds between alginate chains, resulting in a stable polymer network with superior mechanical integrity. Unlike ionic gelation, these networks are resistant to ion exchange and physiological dissolution. The most common method is carbodiimide chemistry, where reagents such as 1-ethyl-3-(3-dimethylaminopropyl)carbodiimide (EDC) and *N*-hydroxysuccinimide (NHS) activate the carboxyl groups on the alginate backbone [29]. This activation facilitates the formation of stable amide bonds when reacted with bifunctional crosslinkers like adipic acid dihydrazide (AAD), as illustrated in Figure 4.

Furthermore, click chemistry has emerged as a premier strategy for achieving high specificity and precise control over crosslinking density. A concrete example is the strain-promoted azide–alkyne cycloaddition (SPAAC), utilizing dibenzocyclooctyne (DBCO)-functionalized alginate and azide-terminated crosslinkers. This bioorthogonal approach allows for rapid, catalyst-free gelation under physiological conditions, ensuring that functional group distribution is highly predictable. While these covalent methods produce mechanically robust gels that do not undergo ion exchange, the synthesis of functionalized precursors often requires meticulous purification and precise control of reaction conditions compared to simple ionic gelation [30].

### 3.3. Dual Crosslinking

**Figure 5:**
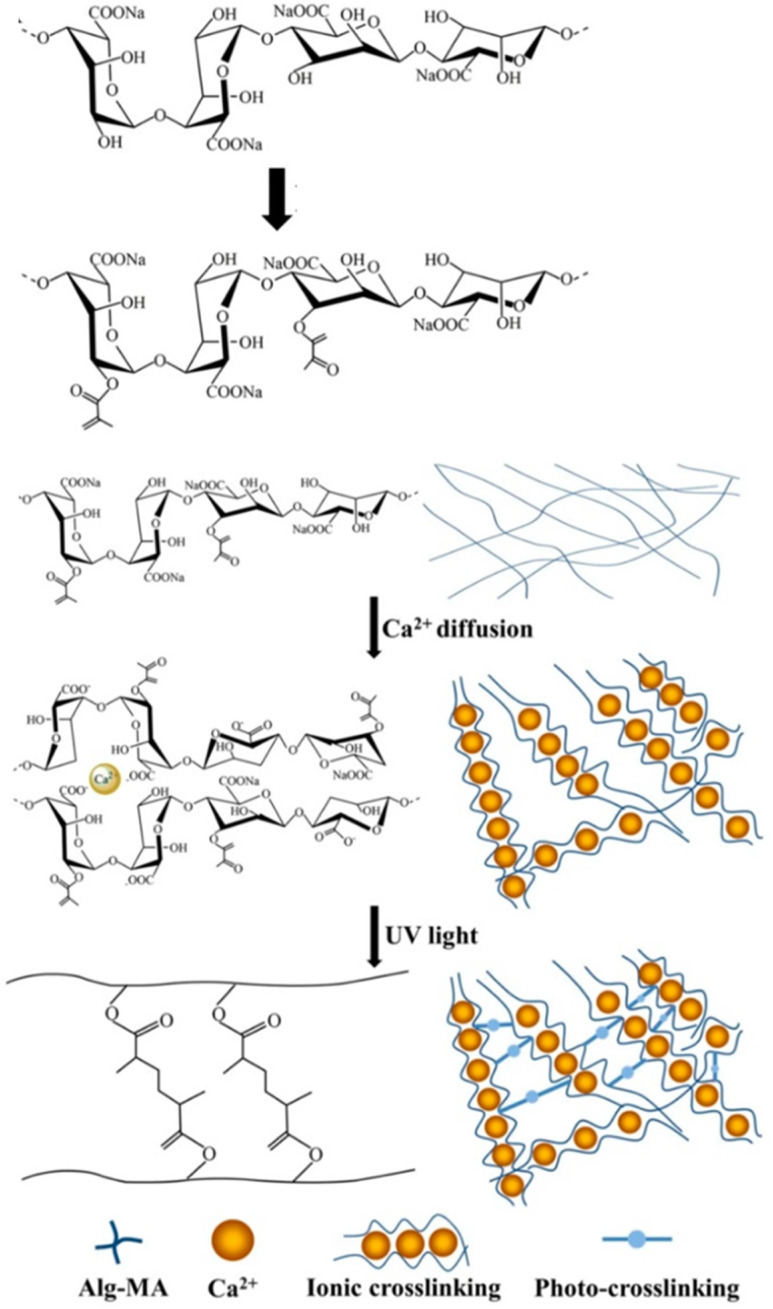
Schematic illustration of dual crosslinking of Alg-MA gels. Image reprinted from ref. [33].

Dual crosslinking is a method that combines both ionic and covalent crosslinking to form gels with enhanced mechanical properties [28]. The main advantage of this method is the immediate gelation and formation of a stable gel that is ideal for injectable applications. The formation of covalent bonds allows for long-term stability and resistance to ion exchange. The dual method produces gels with balanced elasticity and durability, which makes them suitable for load-bearing applications in tissue engineering. The type of crosslinking affects the biocompatibility and stability of alginate gels, and this was demonstrated using alginate microcapsules for islet transplantation [31,32]. Doubled-crosslinked methacrylate alginate (Alg-MA) hydrogel microfibers fabricated by the microfluidic fabrication system were effective in controlling the fracture of macromolecular chains (Figure 5). The microfibers provided excellent three-dimensional growth and distribution for cells and showed great potential in the development of next-generation scaffolds for regenerative medicine and tissue engineering [33].

## 4. Safety and Immunological Considerations

Alginates for applications in biomedical and food industries should be highly pure and free from residual polyphenols, proteins and endotoxins as these can trigger inflammatory reactions in the body [34]. Appropriate post-extraction refinement methods should therefore be applied to guarantee the purity of alginates. The hydrolysate components of alginates, such as mannuronic acid and guluronic acids, are considered safe and non-toxic components.

The Food and Drug Administration (FDA) has recommended the alginate salts of sodium, potassium, calcium, and ammonium, and derivatives of propylene glycol alginates are generally regarded as safe (GRAS) components for use in oral products [35]. The biocompatibility of alginates has been validated in vivo after nasal [35], ocular [36], oral [36,37], and topical [38] administration. The chemical composition of alginates can significantly affect the immunogenicity property. Otterlei et al. in a study demonstrated that alginates that are rich in M-blocks are more effective in stimulating the production of cytokines than the alginates that are rich in G-blocks [39]. Chronic implantation studies have shown that purified alginate scaffolds are able to integrate into host tissue without any systemic toxicity [40].

## 5. Enhancement of Biodegradability and Biomedical Implications

Alginate has excellent biological performance; however, the enhancement of biodegradation is a desirable property for biomedical applications. Degradation is a critical material property in many biomedical applications, such as drug delivery systems and tissue engineering. The degradation of typical alginate gels is slow and poorly controlled [41,42].

**Figure 6:**
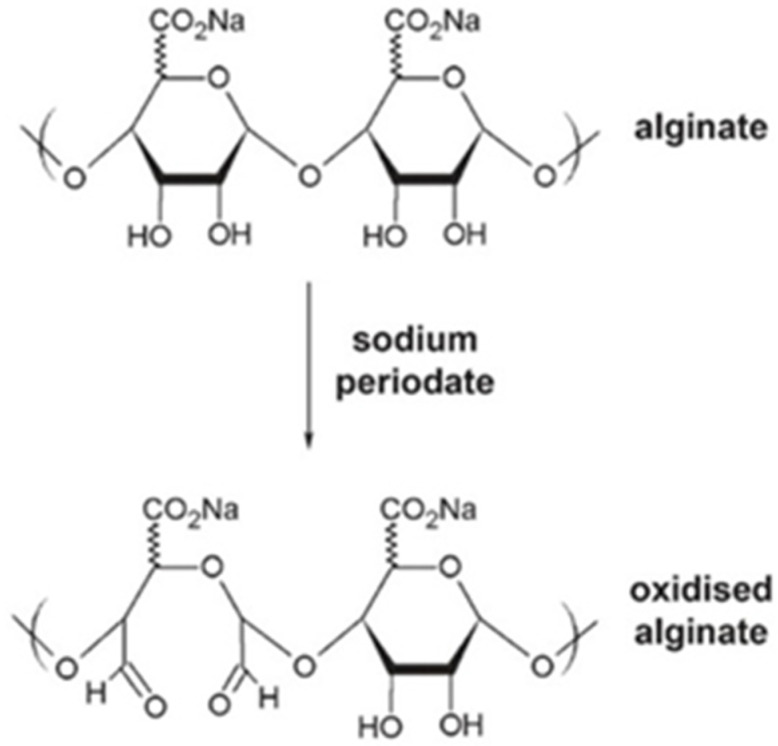
Oxidation of sodium alginate with sodium periodate. Image reprinted from ref. [43].

The biodegradation of alginate can be improved by oxidation with sodium periodate, which breaks the bonds between the carbons of the cis-diol group and converts the chair conformation to an open chain that favours degradation of the polymer backbone [43] (Figure 6). The degradation behaviour depends on the degree of oxidation, pH, and temperature. The oxidation process does not affect the ionotropic crosslinking ability of the alginate to form gels.

Hydrogels based on poly(aldehyde guluronate) prepared from alginate by acid hydrolysis and oxidation followed by crosslinking with adipic acid dihydrazide were highly biodegradable in aqueous media. The hydrolysis of hydrazone bonds between the aldehyde group of poly(aldehyde guluronate) and the hydrazide of adipic acid dihydrazide facilitated the degradation [44]. By combining partially oxidized polymer chains of alginate and a bimodal distribution of molar mass, the degradation of alginate gels can be regulated [44]. The rate of oxidation not only modifies the biodegradability of alginate gels but also their cytotoxicity. Biodegradable photo-crosslinked alginate gels formed by oxidation and methacrylate of alginate did not exhibit any cytotoxic effects in human bone marrow-derived mesenchymal stem cells [45].

Oxidized alginate gels containing collagen (type IV) showed increased viability of corneal epithelia cells, and partially oxidized gels promoted the generation of cartilage-like structures [46]. The degradation of alginate gels can further be improved by suitable encapsulation of fibroblasts [47]. Addition of sulphur to alginate gels through the process of sulfation is another method to improve degradability and to reduce cytotoxicity. Stronger binding of growth factors and anticoagulant activity are achieved through sulfation of alginate gels as coatings for vascular grafts [48]. Grafting of bioactive molecules on alginate such as integrin, tri-peptides, and poly(ethylene glycol) improves cell adhesion and proliferation, which are important for localized drug delivery applications [49].

Incorporation of nanomaterials such as hydroxyapatite, graphene oxide, and metallic nanoparticles improves the mechanical strength of alginate gels and introduces new properties such as osteoconductivity and antimicrobial activity that are vital for biomedical applications. Alginate gels have been modified by physical, chemical or biological methods to improve their physicochemical, mechanical and biological properties for targeted biomedical applications, and a few of these modifications are summarised in Table 1.

## 6. Biomedical Applications of Alginate Gels

### 6.1. Drug Delivery and Encapsulation Systems

**Figure 7:**
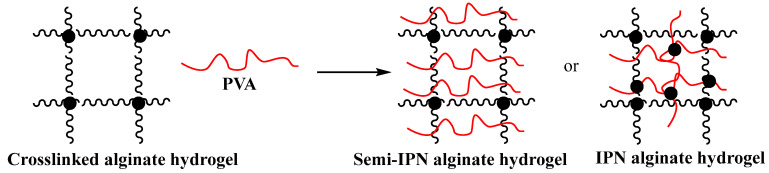
Schematic illustration of alginate gel and formation of IPNs. Image modified from ref. [60].

Encapsulation capability is a key feature for the success of any controlled and targeted drug delivery system, as the therapeutic molecules need to be protected during their transport through the gastrointestinal tract [59]. Alginate forms gels under mild conditions, making it an excellent candidate for drug delivery applications, and the material can be made into microspheres, beads, liposomes, nanoparticles and nanofibers. Alginate gels with either semi- or complete interpenetrating polymer networks (IPNs) have been developed using poly(vinyl alcohol) (PVA) (Figure 7). Such materials exhibited improved mechanical properties and sustained release of the encapsulated drugs over a period of 24 h [60].

Polyelectrolyte complexes of alginate with chitosan in the form of nanoparticles were effective in the encapsulation of a variety of drugs and their cellular uptake [61]. Drugs of small molar mass such as pain killers and antibiotics have been encapsulated successfully in alginate gels. Alginate gels containing diclofenac sodium (a painkiller drug) were effective in targeted release of the drug (95%) in the intestine, with minimal release in the stomach [62]. Oral administration of insulin is challenging due to degradation in the gastrointestinal tract. Microparticles were developed based on alginate and insulin by an internal gelation and emulsification process with an encapsulation efficiency of 80%. The formulation provided enhanced transportation and release of insulin in the intestine with minimal enzymatic degradation. Addition of non-ionic surfactants to the microparticle formulation provided stability of insulin against the pH conditions of the gastrointestinal tract [63]. Various types of alginate gels have been developed as nanocarriers for targeted delivery of anti-cancer drugs to the affected sites in the body, and a few important systems are summarized in Table 2.

Effective uptake in 4 T1 breast cancer cells has been demonstrated in alginate nanoparticles and, in diabetes treatment, alginate-based oral insulin delivery is a non-invasive alternative to injections [64]. The alginate in combination with chitosan or polyurethane provides enhanced mucoadhesion and bioavailability [65]. Enzyme-triggered and pH-responsive alginate and chitosan-based nanoparticles containing curcumin-cyclodextrin complexes significantly reduced colitis inflammation in mice, thereby showing promise in colon-targeted drug delivery [66]. Alginate-based transdermal systems containing curcumin or gabapentin/acetaminophen provide enhanced drug permeation and prolonged therapeutic effects [67].

### 6.2. Wound Healing

**Figure 8:**
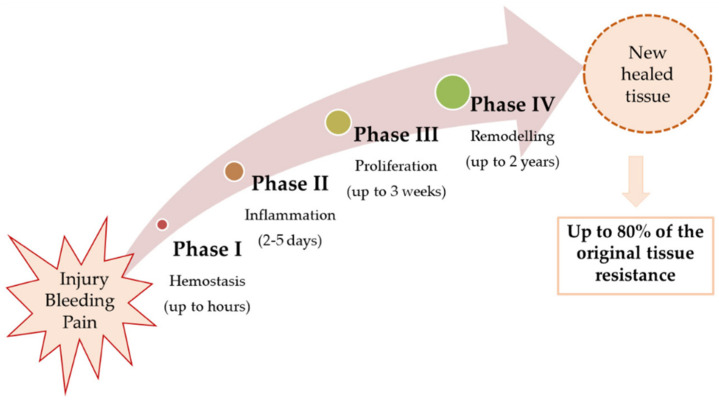
Illustration of the various phases of wound healing. Reprinted from ref. [67].

Wound healing is a complex multi-phase process with four overlapping stages: (i) haemostasis, (ii) inflammation, (iii) proliferation, and (iv) remodelling, as shown in Figure 8. Each stage of wound healing involves blood clotting, immune defence, tissue formation and tissue strengthening to restore the integrity of the skin [68].

**Figure 9:**
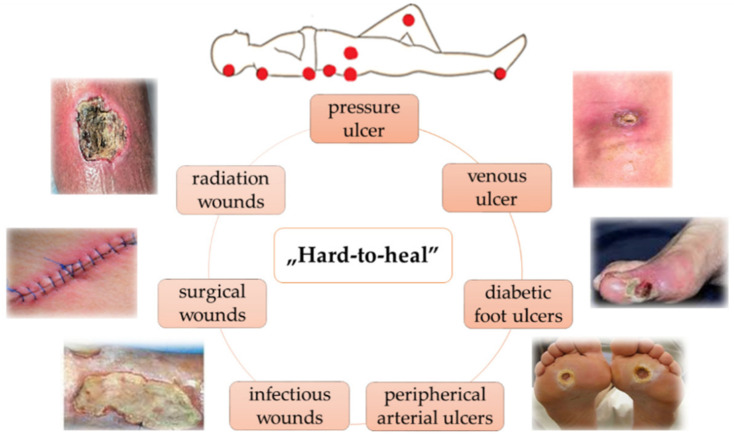
Illustration of common chronic wounds based on WHO classification. Reprinted from ref. [67].

Diabetes can disrupt the normal wound healing process and lead to the formation of chronic wounds such as diabetic foot ulcers, venous ulcers, pressure sores, radiation wounds and infectious wounds, as shown in Figure 9. The chronic wounds are difficult to manage due to the generation of excess exudates, impaired circulation and infections [67,68,69].

To overcome the limitations associated with wound healing, various types of polymeric biomaterials have been developed to support the healing process. The properties of alginate, such as biocompatibility, swelling and absorption, make it suitable for applications in wound dressings, particularly for chronic and burn wounds [70]. These dressings promote wound healing due to their moisture retention properties. The alginate fibres absorb wound exudate, and during this process, the calcium ions within the dressing are exchanged with sodium ions in the wound exudate, thereby providing a cooling and cohesive effect [71]. The moist environment promotes re-epithelialization, stimulates the migration of fibroblasts and keratinocytes, and allows autolytic debridement of necrotic tissue. In addition to providing a moist environment, alginate gels are non-adherent and reduce pain and trauma during the removal of wound dressings.

**Figure 10:**
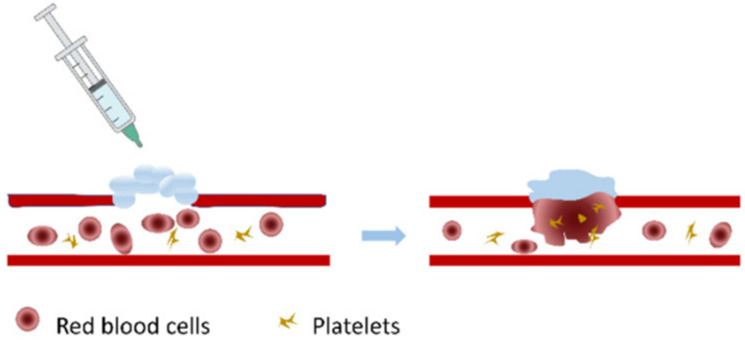
Schematic representation of alginate-based wound dressing in the form of injectable gel promoting hemostasis. Reprinted from ref. [72].

Another important feature of alginate dressings in the form of injectable gel is that it promotes hemostasis, which is vital for wound healing. The calcium ions released from the alginate allow platelet aggregation and activate the clotting factors at the site of the wound, as illustrated in Figure 10. This type of dressing is beneficial for post-surgical wounds and acute trauma. In addition, it also provides a physical barrier for microbial infections [72].

Alginate dressings enriched with chitosan, silver nanoparticles, mesoporous silica and honey have been developed and can reduce the risk of secondary infection in chronic or heavily contaminated wounds [73,74]. The commercially available alginate-based wound dressings for the treatment of various types of wounds are shown in Table 3.

Alginate dressings in the form of injectable gels provide a moist environment and allow easy encapsulation and release of therapeutic agents such as antimicrobials, growth factors and stem cells. Further, the injectable materials can be directly injected into irregular wound cavities as they can conform to the wound bed, allowing the development of granulation tissue [77]. Alginate-based wound dressing in the form of nanofiber mats obtained by an electrospinning method resembles the natural extracellular matrix. The nanofibers are porous with high surface area, and these features allow easy exchange of nutrients and therapeutics to the wound. The nanofiber mats allow adhesion and proliferation of fibroblasts and stimulate collagen synthesis, which are critical factors in the development of granulation tissue and wound healing [78]. Alginate-based smart or stimuli-responsive wound dressings are a new generation of wound dressing materials that respond to changes in the microenvironment of the wound, such as pH, temperature and reactive oxygen species (ROS). These materials selectively release the therapeutic load when they detect infection or inflammation in the microenvironment of the wound, thereby minimizing systemic toxicity and maximizing therapeutic efficacy [79].

### 6.3. Tissue Engineering and Regenerative Medicine

**Figure 11:**
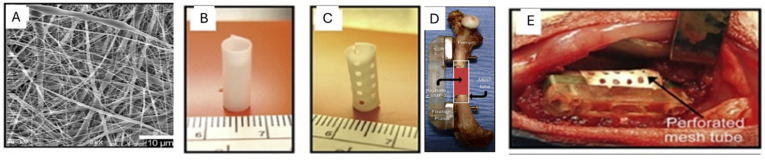
(**A**). Scanning electron microscopy (SEM) image of electrospun nanofiber mesh, showing the smooth and bead-free nano-scaled fibres. (**B**). Hollow tubular implant without perforations constructed from nanofiber meshes. (**C**). Tubular implant with perforations. (**D**). The composite was constructed from an electrospun nanofiber mesh tube applied for repairing the bone defect. (**E**). Picture of the defect after placement of a perforated mesh tube; the alginate inside the tube can be seen through the perforations. Reprinted from ref. [3].

One of the main aims in regenerative medicine is to develop biological substitutes for restoring or enhancing tissue and organ function [80]. Polymeric scaffolds based on alginate have been widely used for the regeneration of various human tissues, as they mimic the extracellular matrix and support cell growth and differentiation. For applications in hard tissues like bones that require good mechanical properties and stability, the alginate gels are often reinforced with bioactive glass or hydroxyapatite nanocrystals to improve mechanical strength and osteogenic activity [81]. Scaffolds based on polyelectrolyte complexes formed between alginate and chitosan exhibited excellent Young’s modulus and improved material–tissue interactions [82,83]. The electrospun nanofiber mesh (Figure 11A) tube containing peptide-modified alginate gel, developed., was effective in cartilage repair and sustained release of growth factors [3]. The hollow tubular implants with and without perforations were constructed from the electrospun nanofiber mesh (Figure 11B,C) and were applied for bone defect repair (Figure 11D,E).

Printed alginate scaffolds with microstructures were effective in improving neuron adhesion and showed promise in neural regeneration. The matrix allowed neurite extension and outgrowth [84]. Alginate-based scaffolds have been developed as artificial niches for mimicking the environment of hematopoietic stem cells. For example, Angiopoietin-I coupled alginate systems can mimic a bone marrow-like environment and preserve the stem cells (LT HSC) in the G0 phase over the long term [85]. A few important alginate-based composite scaffold materials for tissue engineering applications and their properties [84,85,86] are summarized in Table 4.

**Figure 12:**
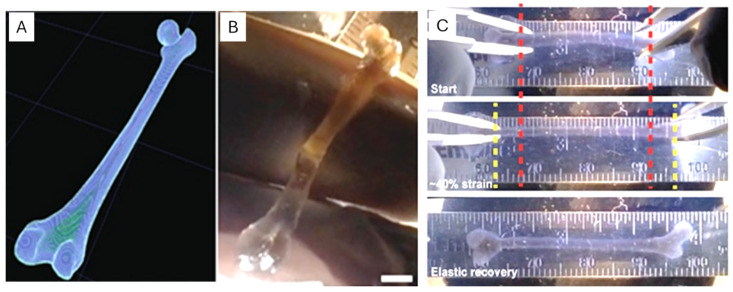
FRESH printing of biological structures based on 3D imaging data and functional analysis of the printed parts [88]. (**A**) A model of a human femur from 3D CT imaging data is scaled down and processed into machine code for FRESH printing. (**B**) The femur is FRESH-printed in alginate, and after removal from the support bath, it closely resembles the model and is easily handled. (**C**) Uniaxial tensile testing of the printed femur demonstrates that it can be strained up to 40% and elastically recover.

3D bioprinting of scaffolds in regenerative medicine has gained much traction in recent years, using alginate as the bioink. The biocompatibility, rheology and ionic crosslinking properties of alginate make it a suitable material for this purpose. These properties allow the encapsulated cells within the scaffold to be protected during the printing process. Various types of functional constructs have been prepared using alginate as bioinks. Alginate inks containing nanocellulose and cartilage have been used to develop patient-specific grafts that support chondrocyte viability up to 90% [87]. Vascularized constructs made using coaxial extrusion with a core containing endothelial/parenchymal cells and a shell containing alginate are perfusable. The existence of the lumen after printing allows for early lumenization, as endothelial cells can form the lining of the construct and allow perfusion [86]. Printing of biological structures (vascular networks, branching airways, and thin-walled cardiac geometries) based on 3D imaging data using freeform reversible embedding of suspended hydrogels (FRESH) has allowed for the preservation of the fine architecture of the final structures. In this type of printing, gelatin microparticles are supported on alginate filaments. FRESH printing of a human femur from 3D CT imaging data and functional analysis of the printed parts are shown in Figure 12 [88].

Recent advances in alginate-based bioprinting focus on improving stability, print fidelity and biological performance. These can be achieved through optimizing the rheology of alginate inks and controlling the ionotropic crosslinking. Step-wise crosslinking, first with ionic gelation using calcium chloride and then by photopolymerization produces stable printed structures of oxidized methacrylate alginate with long-term stability [89]. Alginate bioinks blended with gelatin, gelatin methacrylate or silk fibroin offer greater stability of the 3D printed product and improved cell–matrix interactions [90].

### 6.4. Gastroesophageal Reflux Disease (GERD) Treatment

GERD is a chronic digestive disorder in which stomach acid or bile flows back into the oesophagus, leading to irritation of the oesophagus, heartburn, regurgitation, difficulty in swallowing and chest pain [91]. This happens when the lower oesophagus sphincter becomes weak or relaxes irregularly, allowing the content of the stomach to flow back into the oesophagus. Proton pump inhibitors are commonly prescribed to reduce the production of stomach acid and to promote healing of the oesophagus [92]. The effect of sodium alginate gels containing pectin and cellulose nanocrystals on gastric emptying behaviour has been studied using an in vitro stomach model. Cellulose nanocrystals in the presence of Ca^2+^ and Mg^2+^ ions in gastric conditions form a physical gel with high viscosity, contributing to a slower gastric emptying process [93]. Moayed et al. investigated the dual prophylactic behaviour of alginate gels conjugated with sugar against GERD and *Helicobacter pylori* (*H. pylori*) infection [94]. The gel controlled the acid reflux and significantly reduced the bacterial adhesion to gastric epithelial cells by about 74%. The gel competes with gastric epithelial cells and decreases the amount of *H. pylori* infection in the stomach.

The mechanism of action of alginate in GERD management is through the formation of a gel-like raft that floats on the surface of the stomach content. The gel-like raft acts like a physical barrier and prevents the acidic contents from refluxing into the oesophagus and reduces exposure of the oesophagus lining to stomach acid [95]. The raft gels made with alginate and Aloe vera exhibited improved strength and reflux resistance under simulated gastric conditions because of cohesive and viscoelastic behaviour. The gel suspension also exhibited suitable acid-neutralizing capacity, thereby showing potential application in GERD prevention [96]. A fast-acting gel-raft system based on alginate, polysaccharide and glyceryl monooleate, and alginate-xanthan gum suspension showed improved control of acid reflux, and the effect was comparable to that of commercially available Gaviscon [97,98].

### 6.5. Management of Diabetes

According to the World Health Organization (WHO), by 2030 diabetes will be the 7th leading cause of death globally. If the disease is not well managed, diabetes-related micro and macrovascular complications such as renal failure, heart attacks, stroke, blindness and lower limb amputations may occur [99]. One of the main treatments for diabetes is administration of insulin through injections, and non-invasive oral administration has been widely studied by pharmaceutical industries. However, oral administration of insulin faces several barriers, such as enzymatic hydrolysis and first-phase metabolism in the gastrointestinal tract. To overcome these barriers, encapsulation strategies have been explored for targeted delivery of insulin. Drug delivery systems consisting of sodium alginate, chitosan and insulin showed improved blood glucose and the bioavailability of insulin by 8.11%. The polymer combination tolerated the acidic pH of the stomach and did not exhibit any toxic effects during in vitro and in vivo studies [100].

Nanoparticles of alginate encapsulated with insulin are a promising system for potential oral delivery of insulin with good stability and strong absorption capacity. The small size of the system allows high loading and protection against proteolytic enzymes, enhanced mucoadhesion and increased retention [101]. Alginate coating of antidiabetic (hypoglycemic) drugs counteracts blood glucose level fluctuations and is effective for the prolonged and sustained delivery of the drugs [102].

### 6.6. Management of Obesity

Obesity is a complex multifactorial disease resulting from various factors such as genetic, epigenetic, behavioural, physiological, and socio-cultural factors. Management of obesity requires dietary modifications and a disciplined lifestyle. Dietary fibre consumption is associated with weight loss, and a number of clinical trials of alternative treatments with dietary fibres have been conducted [103]. Sodium alginate, due to its anti-inflammatory and postprandial blood glucose-reducing properties, is marketed as a weight loss supplement either as a standalone product or as a food additive. Capsules made of sodium alginate dissolve and swell into a soft gel in the gastric environment, remain stable for 6 to 8 h and then dissolve when they reach the intestine [104]. The presence of soft gel in the stomach increases satiety and inhibits the gastrointestinal enzymes, thereby lowering glucose and cholesterol absorption. Interestingly, when added to food as a stabilizer (E401), the above effects are minimal due to the low concentration of sodium alginate. Alginate is widely incorporated in various food substances such as bread, cereal bars and beverages due to its triglyceride-lowering properties [105].

**Figure 13:**
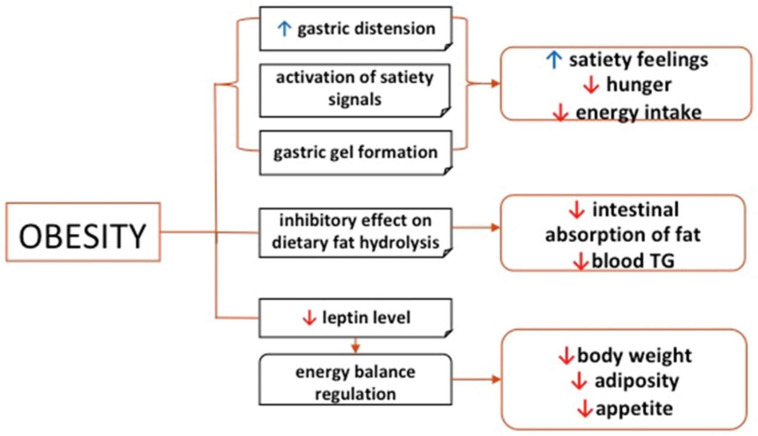
Health benefits of alginate and possible mechanisms of action in the management of obesity. Reprinted from ref. [99].

The pH-dependent interactions between alginate and protein prevent the action of pepsin without affecting the action of trypsin. At low pH, alginate binds with pepsin and removes it by precipitation, thereby preventing the enzyme-substrate interaction [106]. The physicochemical properties of alginates are affected by the ratio of M and G-blocks in the materials. Alginates with a high content of G-blocks interact with glycoproteins and increase fatty acid excretion, which plays a major role in weight management [107]. G-block-rich alginate exhibits higher inhibitory capacity for lipase than alginate that is rich in M-blocks, thereby making alginate an attractive option for the control of dietary lipids. The exact mechanisms by which alginate acts on the food intake pathways and regulation of body weight are not fully understood. Nevertheless, evidence from various studies [102,103,104,105,106,107,108] suggests that alginate is a promising candidate in the management of obesity, and the overall effects and possible mechanism of action of alginate are illustrated in Figure 13 [99].

### 6.7. Clinical Translation and Marketed Products

Many products that are based on alginate have been translated into clinical applications and into the market [109]. These products are available for dermal, oral, and rectal delivery of therapeutics, and a few important pharmaceutical products are presented in Table 5.

## 7. Challenges

Alginate-based materials face several important limitations that continue to restrict their broader application. A major weakness is their poor mechanical strength, as alginate hydrogels are often soft, brittle, and unable to withstand prolonged mechanical stress, particularly in load-bearing environments. They also tend to undergo excessive swelling in aqueous media, which can alter their shape, reduce structural stability, and affect the controlled release of incorporated agents [110].

Another key limitation is their instability under physiological conditions. Ionically crosslinked alginate, especially calcium-alginate, is vulnerable to ion exchange with surrounding fluids, which can weaken the gel network, accelerate disintegration, and compromise long-term performance. Their degradation profile is also difficult to control, as some formulations degrade too rapidly while others persist longer than desired. In biological applications, alginate has limited intrinsic bioactivity because it lacks natural cell-adhesion sites, meaning that many mammalian cells do not readily attach, spread, or proliferate on pure alginate surfaces. This often necessitates additional chemical modification or blending with other materials, which increases complexity. Another limitation is batch-to-batch variability, since alginate is derived from natural sources and its molecular weight, purity, viscosity, and mannuronic/guluronic acid ratio can vary significantly, directly influencing gelation behaviour and final material properties [111].

Residual contaminants from extraction may also affect reproducibility and biocompatibility. Sterilization presents an additional challenge, as some methods can alter viscosity, crosslinking behaviour, or overall material performance. Furthermore, achieving precise control over porosity, drug-loading efficiency, and sustained release behaviour remains difficult in many alginate systems. From a translational perspective, scale-up and standardization are still problematic, particularly when high reproducibility is required for biomedical use. These limitations highlight that, despite its advantages, alginate still requires substantial optimization to improve its strength, stability, bifunctionality, and consistency [112].

## 8. Conclusions and Future Perspective

Alginate remains a premier biomaterial in both biomedical and nutritional science, underpinned by its exceptional biocompatibility and the predictable crosslinking defined by the “egg-box” model. By overcoming historical limitations regarding purity and mechanical strength, alginate-based systems have successfully transitioned from laboratory research to widely utilized commercial products, including advanced wound dressings, tissue scaffolds, and anti-reflux suspensions. These applications rely on the structural-functional relationship inherent to the polymer’s architecture, which provides the mechanical stability necessary for effective clinical management.

While the foundation of alginate in classical biomedical applications is robust, its role in the management of obesity and diabetes represents a significant clinical frontier. Alginate gels offer a strategic, non-pharmacological pathway for metabolic support, acting as biophysical barriers to nutrient absorption and mechanical triggers for satiety. Future research should prioritize the integration of precisely crosslinked alginate into functional food systems designed to modulate caloric intake and glycemic response. However, to fully realize this potential, further investigation is required into the long-term physiological impact of these matrices within the gastrointestinal environment. By refining the synchronization between “egg-box” chemistry and human physiology, alginate-based technologies are poised to provide sophisticated, material-based solutions for global metabolic health challenges.

## Figures and Tables

**Table 1:** Summary of modification of alginate gels with additives for enhanced properties.

Method	Additives	Properties	Ref.
Physical	Aloe vera, polyvinyl alcohol	Thermal stability; improved release.	[50]
Physical	Borax, fibronectin-binding integrin	Increased cell adhesion; improved myofibre fusion; enhanced muscle regeneration.	[51]
Physical	Probiotics	Increased bioavailability; improved stability under high temperature and dehydration.	[52]
Chemical	Chitosan	Protein-trapping capability; controlled biodegradation; sustained drug delivery profile.	[53]
Chemical	Poly(lactic acid), curcumin	Hemocompatibility; cytocompatibility; antibacterial.	[54]
Chemical	Polyethylene glycol	Improved mechanical and thermal properties.	[55]
Chemical	Hyaluronic acid, poly(*N*-isopropyl acrylamide), chitosan	Improved thermal and cell-adhesion properties.	[56]
Enzymatic	Enzymatically derived chitosan derivatives	Enhanced biocompatibility and cytotoxicity.	[57]
Other	3D-printing of alginate scaffolds	Improved mechanical and biological properties.	[58]

**Table 2:** Alginate gels as nanocarriers of anti-cancer drugs [61,62,63].

Type of Nanocarrier	Composition and Size (nm)	Drugs	Properties
Nanoparticles	Alginate/chitosan (212–552)	Curcumin glutaric acid	Enhanced cellular uptake
	Alginate/chitosan (~80)	Doxorubicin	Enhanced cellular uptake
	PLGA/alginate/chitosan (>200)	Doxorubicin	
	Alginate/chitosan (115)	5-aminolevulinic acid	Folic-acid receptor-based endocytosis
	Alginate (274)	Doxorubicin	Glycyrrhizin acid-mediated endocytosis
	Alginate, hydroxyapatite, iron oxide (9.6–20)	Curcumin and 6-gingerol	pH-responsive
	Alginate, mesoporous silica (~100)	Doxorubicin	pH and redox responsive
	Alginate/chitosan/MnFe_2_O_4_ (~200)	Curcumin	Magnetic responsive
Nanogels	Alginate/cyclodextrin (55.1)	5-flurouracil	Pressure responsive
	Alginate/keratin (~100)	Doxorubicin	GSH/trypsin responsive
Micelles	Alginate-g-PNIPAm (30–300)	5-flurouracil	pH/temperature responsive
	Alginate-curcumin (200)	Curcumin	Enhanced cellular uptake
	Alginate-curcumin (235)	Curcumin	ASGPR-mediated endocytosis
Nanodroplet	Alginate (551)	Doxorubicin	Ultrasound responsive
Nanohybrid	Alginate-doxorubicin (142)	Doxorubicin	pH responsive
Nanocomplex	Alginate/PNIPAM/chitosan	Doxorubicin/temozolomide	Folic-acid receptor-based endocytosis

PLGA: poly(lactic glycolic acid), PNIPAm: poly(*N*-isopropylacrylamide).

**Table 3:** A few important commercially available alginate-based wound dressings [75,76].

Product Name	Composition	Applications
Algicell^TM^	Sodium alginate and silver (1.4%)	Diabetic foot ulcers, leg ulcers, pressure ulcers, traumatic and surgical wounds.
AlgiSite M^TM^	Calcium alginate	Leg ulcers, diabetic foot ulcers and surgical wounds.
Comfeel Plus^TM^	Calcium alginate and sodium carboxymethyl cellulose	Venous leg ulcers, burns, pressure ulcers, surgical and necrotic wounds.
Kaltostat^TM^	Sodium alginate	Pressure ulcers, venous ulcers, diabetic foot ulcers and traumatic wounds.
Sorbsan^TM^	Calcium alginate	Arterial, venous, pressure and diabetic foot ulcers. Donor and graft sites, and traumatic wounds.
Tegagen^TM^	Sodium alginate	Diabetic and infected wounds.
Guardix-SG^®^	Sodium alginate and poloxamer	To avoid post-operative adhesions in thyroid and spine surgeries.
SeaSorb^®^	Calcium alginate	High exuding wounds.
Algivon^®^	Calcium alginate and Manuka honey	Eliminates odour and ideal for necrotic wounds.
Fibracol^TM^ Plus	Calcium alginate and collagen	For full and partial thickness wounds, diabetic foot ulcers and second-degree burns.
Hyalogran^®^	Sodium alginate and ester of hyaluronic acid	Diabetic foot ulcers, ischemic and necrotic wounds.
Tromboguard^®^	Sodium alginate, calcium alginate, chitosan, polyurethane and silver ions	To stop bleeding in surgical wounds, gunshot and traumatic wounds.

**Table 4:** Alginate-based composites in tissue engineering applications.

Composite	Properties	Application
Alginate/collagen	Superior cell adhesion, strong mechanical strength, increase expression of cartilage-specific genes	Cartilage tissue engineering
Alginate/bioactive glass	Improved osteogenic differentiation, good mechanical strength and bioadhesion	Bone tissue engineering
Alginate/chitosan/flurbiprofen	Excellent mechanical, hydrophilic and anti-inflammatory properties	Skin tissue engineering
Alginate-Ga-based glass	Enhanced mechanical properties and biocompatibility	Cardiovascular tissue engineering
Alginate/polycaprolactone/carboxymethyl chitosan	Excellent osteoconductive capacity, biocompatibility and mechanical properties	Periosteal tissue engineering

**Table 5:** Important commercially available alginate-based pharmaceutical products [109].

Product	Manufacturer	Administration	Main Ingredients	Indications
Algivon^®^ dressing	Advancis Medical (Kirkby-in-Ashfield, United Kingdom)	Dermal	Calcium alginate and Manuka honey	Necrotic and malodorous wounds
ChondroArt 3D^®^ injection	Arkopharma (Carros, France)	Arthroscopic	Alginate, agarose and autologous chondrocytes	Osteochondrosis and osteoarthritis
Gaviscon^®^ Double Action Liquid	Reckitt Benckiser Healthcare (Berkshire, United Kingdom)	Oral	Sodium alginate, calcium carbonate, and sodium bicarbonate	Acid reflux
Natalsid^®^suppositories	STADA (Bad Vilbel, Germany)	Rectal	Sodium alginate	Chronic haemorrhoids, proctosigmoiditis and chronic anal fissures
Progenix putty^®^	Medtronic Spinal & Biologics (Dublin, Ireland)	Periodontal	Sodium alginate, and type-1 bovine collagen	Bone gaps and voids
Purilon Gel^®^	Coloplast (Humlebaek, Denmark)	Dermal	Calcium alginate, and sodium carboxymethyl cellulose	Necrotic and sloughy wound, and first- and second-degree burns

## Data Availability

The data is available with the corresponding author.
